# Dengue 2 serotype and yellow fever coinfection

**DOI:** 10.1099/acmi.0.000300

**Published:** 2021-12-17

**Authors:** Flávia Gehrke, Katharyna Cardoso Gois, Beatriz da Costa Alves Aguiar Reis, Gabriel Zorello Laporta, Fernando Luiz Affonso Fonseca

**Affiliations:** ^1^​ Programa de Pós Graduação em Ciências da Saúde, Instituto de Assistência Médica ao Servidor Público Estadual (IAMSPE), São Paulo, Brazil; ^2^​ Patologia, Centro Universitário FMABC, Santo André, São Paulo, Brazil; ^3^​ Programa de Pós Graduação em Ciências da Saúde, Centro Universitário FMABC, São Paulo, Santo André, Brazil; ^4^​ Laboratório de Análises Clínicas, FMABC-Centro Universitário Saúde ABC, Santo André, São Paulo, Brazil; ^5^​ Departamento de Ciências Farmacêuticas, Universidade Federal de São Paulo (UNIFESP), Diadema, São Paulo, Brazil; ^‡^​ Present address: Programa de Pós Graduação em Ciências da Saúde, Centro Universitário FMABC, São Paulo, Santo André, Brazil

**Keywords:** coinfection, arbovirus infections and diagnosis

## Abstract

**Case Presentation:**

Arboviruses primarily consist of RNA, which favours greater genetic plasticity, with a higher frequency of mutations that allow the virus to adapt to different hosts. The initial symptomatology is nonspecific, in that the patient can present fever, myalgia, arthralgia, rash and headache. This makes a clinical diagnosis using laboratory tests difficult and time-consuming. In Brazil, the main arboviruses involved in epidemics belong to the family *Flaviviridae*. The patient in this case is from the municipality of São Bernardo do Campo, an area endemic for arboviruses. He presented symptoms of fever, myalgia and headache.

**Results:**

The multiplex assay for arboviruses detected genetic material from the dengue 2 and yellow fever viruses.

**Conclusion:**

This result confirms the importance of molecular tests showing high sensitivity and specificity that can assist clinical diagnosis, particularly in endemic areas during periods of outbreak for other arboviruses, like the epidemiological picture in Brazil in 2018, when significant co-circulation of dengue virus and yellow fever virus occurred. The presence of co-circulating arboviruses increases the chance of coinfection and demonstrates the importance of differential diagnosis.

## Introduction

Arboviruses primarily consist of RNA, which favours greater genetic plasticity, with a higher frequency of mutations that allow the virus to adapt to different hosts [1]. Transmission mainly occurs during blood meals imbibed by infected arthropods, but can occur during blood transfusion and congenital transmission, and there have even been some reports of sexual transmission [1–3]. The initial symptomatology is nonspecific, in that the patient can present fever, myalgia, arthralgia, rash and headache. This makes a clinical diagnosis using laboratory tests difficult and time-consuming [1]. Sensible and specific methodologies can contribute to differential diagnosis.

In Brazil, the main arboviruses involved in epidemics belong to the family *Flaviviridae* [1]. There are four serotypes of dengue virus (DENV) in co-circulation, which favours hyperendemicity. In 2018, in Brazil, 174 724 DENV cases were confirmed, and of these, 155 (0.08 %) died. The incidence rate was 127.5 cases/100.000 inhabitants and the most common serotype was DENV2 [4]. In the State of São Paulo, 14 206 cases were confirmed, while in the municipality of São Bernardo do Campo, 7 cases of DENV were confirmed in 2018 [5, 6].

In the State of São Paulo, 538 cases of yellow fever (YF)were confirmed, resulting in 184 (34.2 %) deaths [7]. The most critical situation was observed in the first trimester of 2018, between January and March, when 79 patients with laboratory-confirmed yellow fever were admitted to the intensive care unit (ICU) in a tertiary hospital in Sao Paulo because of rapid clinical deterioration; 53 (67 %) patients went on to die [8].

The laboratory diagnosis of flavivirus is complex. Rapid tests show sensitivity between 40–70 %, while those using the ELISA methodology have sensitivity up to 95 %, although these tests show greater cross-reactivity [9]. In addition, it has already been demonstrated *in vitro* that serum samples from convalescent patients for dengue have demonstrated neutralizing activity against yellow fever virus (YFV) (55.6 %) and St Louis encephalitis virus (SLEV) (33.4 %). Serum samples from patients convalescent for Zika virus (ZIKV) were able to neutralize West Nile virus (WNV) (40 %), Ilheus virus (ILHV) (40 %) and Rocio virus (ROCV) (20 %), confirming cross-reactivity between sera and difficulty in serological diagnosis [10].

YFV is epidemiologically important due to its clinical severity and its potential for dissemination in urban areas infested by the mosquito *Aedes aegypti*. In the last two decades, YFV transmissions were registered outside the limits of the area considered to be endemic (the Amazon region).

## Case report

The patient is from the municipality of São Bernardo do Campo and was admitted to a public hospital of the same city in July 2018, showing clinical symptomatology compatible with an arbovirus: fever, myalgia and headache.

Biochemical, serological and molecular tests were requested to detect arboviruses. Serum RNA was extracted using the QIAamp Viral RNA Mini kit (Qiagen, Hilden, Germany), and the RNA was then converted into cDNA using the QuantiNova Reverse Transcription kit (Qiagen, Hilden, Germany) and multiplex qRT-PCR composed of two platforms to determine the virus (platform A: four viral serotypes of dengue, yellow fever, Zika, chikungunya; and platform B: Mayaro, Rocio, St Louis and West Nile) was performed in an ABI 7500 thermal cycler. Primers and probes were designed with the assistance of the IDT First Design program (PrimerQuest program, IDT, Coralville, IA, USA).

Laboratory tests revealed a leukocytosis of 20.9 103 mm^−3^, an increase in direct bilirubin of 0.9 mg dl^−1^, alanine aminotransferase (ALT) of 68 μ l^−1^, aspartate transaminase (AST) of 60 μ l^−1^, gamma-glutamyl transferase (GGT) of 301 μ l^−1^ and C-reactive protein (C-PR) of 240.59 mg dl^−1^ (Table 1). Haemoculture and urine cultures were both negative. Serology for hepatitis B, C and HIV I and II were all negative, while IgG was positive for toxoplasmosis, cytomegalovirus and Epstein–Barr virus. Rapid antibody tests for dengue (IgM and IgG) and viral protein NS1 were negative, while the test for YFV IgM was inconclusive. In multiplex qRT-PCR, genetic material from DENV2 and YFV was detected.

**Table 1.** Laboratory findings for patient during hospital stay

Characteristics	Maximum	Minimum	Reference values
**Blood routine**			
Erythrocytes	4.97	4.01	4.5–5.5 million mm^−3^
Haemoglobin	13.7	11.1	13–17 g dl^−1^
Haematocrit	40.8	32.2	40–50 %
Leukocytes	25.7	4.4	4.5–11 million mm^−3^
Neutrophils	22.86	2.65	2.0–8.00 million mm^−3^
Eosinophils	0.45	0.11	0–0.40 million mm^−3^
Basophils	0.03	0.02	0–0.2 million mm^−3^
Lymphocytes	3.76	0.66	1.00–5.00 million mm^−3^
Monocytes	1.05	0.18	0.10–1.0 million mm^−3^
Platelets	352	165	150–400 million mm^−3^
**Blood biochemistry**			
Total bilirubin	1	0.4	≤1.2 mg dl^−1^
Direct bilirubin	0.9	0.3	≤0.2 mg dl^−1^
Indirect bilirubin	0.3	0.1	≤0.8 mg dl^−1^
Creatine phosphokinase	67	41	<190 μ l^−1^
Alkaline phosphatase	175	117	40–129 μ l^−1^
Gama glutamyl transferase	518	301	8–61 μ l^−1^
Alanine aminotransferase	68	40	≤41 μ l^−1^
Aspartate transaminase	60	23	≤40 μ l^−1^
**Infection-related biomarkers**			
C-reactive protein	321.74	117.83	≤5 mg dl^−1^

The patient was hospitalized for 15 days, during which time he was given 0.9 % saline and 1.0 g of novalgine; hepatic enzyme stabilization occurred and clinical recovery was observed; there was no sequel.

## Discussion

This result confirms the importance of molecular tests showing high sensitivity and specificity that can assist clinical diagnosis, particularly in endemic areas during periods of outbreak of other arboviruses, like the epidemiological picture in Brazil in 2018, when significant co-circulation of DENV and YFV occurred. The presence of co-circulating arboviruses increases the chance of coinfection and demonstrates the importance of differential diagnosis and vector control.

In a study in Bali involving travellers who showed at least one clinical symptom for dengue, laboratory tests showed that 66.2 % were contaminated with DENV. In this same study they were unable to detect Japanese encephalitis, Zika and chikungunya, indicating that other viruses are circulating in that location [11].

A study of 652 patients in the Democratic Republic of Congo detected 3 cases of co-infection by DENV1 and 2 using qRT-PCR. The data obtained indicated the importance of molecular differential tests in directing health actions [12].

Dengue is the disease with the highest incidence in travellers, and the relative risk of severe dengue in the second infection can increase by sevenfold [13]. In addition, in places where the disease is not endemic, it is important that human and vector surveillance systems take effective measures in imported cases to prevent the occurrence of indigenous cases [14].

In addition, a country’s socio-economic conditions can cause a large influx of immigrants from endemic areas to other countries. The search for better living conditions for immigrants from countries in the African and South American continents may incur an increase in cases of some arboviruses [15]. Hence, testing travellers and migrants could be an optional tool and would also increase surveillance in the globalization of diseases.

Broad differential testing for arboviruses has a relevant epidemiological role, as carried out in Colombia between 2015–2016, during the ZIKV epidemic. Molecular tests were performed on 23871 samples that detected 1.84 % DENV, 1.07 % CHIKV and 42.38 % ZIKV, with 0.14 % CHIKV–ZIKV coinfection cases [16].

The use of two molecular platforms to investigate eight viral types has been shown to be effective and useful, given that Brazil is an endemic country for DENV and there are several different arboviruses in co-circulation. This is especially important, since these arboviruses present similar clinical features and laboratory tests are required to achieve a diagnosis.

The proposed platforms were capable of detecting the arboviruses studied. It is important that such tests increase the detection of different viruses, that the use of such platforms enables rapid laboratory diagnosis, the detection of endemic viruses, viruses involved in recent outbreaks and viruses of low incidence when routine tests are negative.
